# Editorial: Place-based evidence for clinical artificial intelligence implementation

**DOI:** 10.3389/frhs.2025.1736656

**Published:** 2025-11-26

**Authors:** Henry David Jeffry Hogg, Janak Gunatilleke, Gregory Maniatopoulos

**Affiliations:** 1 Department of Applied Health Sciences, School of Health Sciences, College of Medicine and Health, University of Birmingham, Birmingham, United Kingdom; 2 University Hospitals Birmingham NHS Foundation Trust, Birmingham, United Kingdom; 3 KPMG, London, United Kingdom; 4 University of Leicester, Leicester, United Kingdom

**Keywords:** frameworks, evidence - based medicine, implementation, artificial intelligence - AI, service evaluation

## Introduction

There has been a sustained and broadly held optimism around the potential of clinical artificial intelligence (AI) to improve on the quality, efficiency and reach of healthcare services. This optimism continues to be signalled by policy makers, manufacturers and researchers (1–3). Despite this general tide of optimism, examples of scaled adoption of specific clinical AI technologies and the forms of evidence traditionally considered most valuable in informing those adoption decisions remain scarce (4). The value of such evidence is challenged by the context-sensitivity of clinical AI's value proposition and the limited availability of skills and guidance to enable local stakeholders to make informed decisions. In addition, challenges are presented by the complexity underpinning the forms of risk that accompany AI's potential benefits (5). Evidence that permits the evaluation of clinical risk is required, as well as evidence highlighting legal, financial, operational and reputational risks. These factors all contribute to the persistent implementation gap around clinical AI and the stubborn but vital challenge of evaluating these interventions in the sociotechnical context in which they are embedded and used (6).

This research topic presents a collection of articles which blur the margins of theory and practice to support decision makers as they evaluate clinical AI interventions for local implementation. The work presented does not seek to directly create the evidence that local decision makers require. This is because of an acceptance that the varied forms of evidence required to demonstrate that the risks of an AI innovation are outweighed by its benefits have limited generalisability beyond the setting in which the evidence was generated. Instead, these articles aim to share generalisable and pragmatic approaches to create that evidence, ensuring place-based meaning. Authors take frameworks from governance, research and industry disciplines and apply varied methodological approaches to produce insights and tools which are actionable for the individuals who are responsible for implementing AI in real-world healthcare services.

In our first article, Nair et al. report findings from secondary research methods and primary qualitative research to explore the activities typical to the implementation of clinical AI. They evaluate and refine an established theoretical framework to provide a roadmap of these activities for healthcare provider organisation. Their analysis highlights the range of individuals, the process and the importance of collective effort in bringing clinical AI into practice whilst showing the enduring relevance of insights from other forms of innovation captured in a theoretical framework. This is followed by Macdonald et al.’s systematic review, which presents a methodology in the form of a Target Product Profile (TPP) designed to facilitate collaboration on clinical AI innovation, not only within a healthcare provider organisation, but also for developers of AI solutions from industry and elsewhere. This work identifies and consolidates TPPs from across digital health technologies to establish a practical framework that enables current and future adopters of clinical AI to signal their needs to developers and to evaluate potential technologies in a holistic and structured way. Our final two contributions offer insights from policy research exploring the challenges and opportunities posed by existent practical frameworks for clinical AI. These frameworks address legal and governance aspects of healthcare innovation which were explored through workshops with cross-sector and multidisciplinary participation. The article from Evans et al. examines procurement frameworks. Recommendations for practitioners in various roles across the healthcare system are presented to unlock the opportunities these frameworks offer, addressing key barriers that hinder the scale and spread of clinical AI innovation. Gilbert et al.’s article shifts the focus from commercial governance to information governance. Drawing upon a combination of clinical, academic, and industry perspectives, the authors reflect on the factors that influence the efficiency and effectiveness of the Data Protection Impact Assessment framework for AI research and practice in the UK, identifying both challenges and potential solutions. In so doing, their analysis highlights the need for training initiatives, communities of practice and the standardisation of governance processes and structures across NHS Trusts.

The above contributions offer diverse approaches to the varied implementation challenges local decision makers face with clinical AI. This encompasses i) methodological diversity (evidence synthesis, qualitative research, co-design), ii) domain diversity (operations, commercial, information governance, innovation) and iii) philosophical diversity (theoretical frameworks, practical frameworks). Collectively, the articles illustrate the interface between implementation research and practice and the potential value of bridging the two. Insights from this topic and related work, both within and beyond academic literature, help to mobilise knowledge from a broader empirical and theoretical base to address the challenge of implementing a clinical AI technology in a specific context. This challenge presents real problems. Significant resources and good will are expended by actors designing and executing pilots in isolation, while their understanding of the requirements and how to evidence success or failure remains limited. The repeated and failed attempts to locally evidence these requirements present a significant threat to the reputation of AI innovations and a waste of scarce resources. In turn, this limited understanding can lead to imprecise estimates of the costs of clinical AI implementation for adopter organisations. A recent Health Technology Assessment estimated the implementation costs for a specific AI intervention for fracture detection on radiographs to vary between £1,200 and £120,000 (7). This lack of precision threatens to completely undermine the viability of decision making under the budgetary constraints of a single department or organisation.

The insights presented here aim to help practitioners in shifting their focus from the novelties of clinical AI to the established knowledge and theory which underpin its successful implementation. This approach supports practitioners in anticipating and managing the challenges of clinical AI implementation, determining what kinds of evidence need to be generated and how that can be done. It does not discard the pursuit of generalisability but focuses on the generalisability of methodologies for place-based evidence generation rather than the evidence itself. In summary, this collection highlights the importance of interdisciplinarity in safe, effective and efficient clinical AI implementation. The articles presented aim to provide practical tools and insights that enable stakeholders within adopting organisations to engage in collective sensemaking to facilitate successful implementation (8).

## References

[1] United States Food & Drug Administration. Artificial Intelligence-Enabled Medical Devices. Available online at: https://www.fda.gov/medical-devices/software-medical-device-samd/artificial-intelligence-enabled-medical-devices (Accessed October 03, 2025)

[2] NHS England. Fit for the Future: 10 Year Health Plan for England. (2025). Available online at: https://www.england.nhs.uk/long-term-plan/ (Accessed October 27, 2025)

[3] Zhang J Whebell S Gallifant J Budhdeo S Mattie H Lertvittayakumjorn P An interactive dashboard to track themes, development maturity, and global equity in clinical artificial intelligence research. Lancet Digit Health. (2022) 4(4):e212–13. 10.1016/S2589-7500(22)00032-2 35337638 PMC9150439

[4] Hogg HDJ Martindale APL Liu X Denniston AK. Clinical evaluation of artificial intelligence-enabled interventions. Invest Ophthalmol Vis Sci. (2024) 65(10):10. 10.1167/iovs.65.10.10 39106058 PMC11309043

[5] Kim JY Boag W Gulamali F Hasan A Hogg HDJ Lifson M Organizational governance of emerging technologies: AI adoption in healthcare. Proceedings of the 2023 ACM Conference on Fairness, Accountability, and Transparency. Chicago, IL, USA: Association for Computing Machinery (2023). p. 1396–417

[6] Hogg HDJ Al-Zubaidy M, Technology Enhanced Macular Services Study Reference Group, Talks J Denniston AK Kelly CJ Stakeholder perspectives of clinical artificial intelligence implementation: systematic review of qualitative evidence. J Med Internet Res. (2023) 25:e39742. 10.2196/39742 36626192 PMC9875023

[7] National Institute for Health and Care Excellence. Artificial intelligence technologies to help detect fractures on x-rays in urgent care: early value assessment. Health Technol Eval. (2025):1–17. https://www.nice.org.uk/guidance/hte20/resources/artificial-intelligence-technologies-to-help-detect-fractures-onxrays-in-urgent-care-early-value-assessment-pdf-50262025025221

[8] Maniatopoulos G Hughes G. Healthcare Innovation, Implementation and Change: A Sensemaking Perspective. London: Palgrave Macmillan (2026). (in press)

